# Prevalence of self-reported diagnosis of diabetes mellitus and associated risk factors in a national survey in the US population: SHIELD (Study to Help Improve Early evaluation and management of risk factors Leading to Diabetes)

**DOI:** 10.1186/1471-2458-7-277

**Published:** 2007-10-03

**Authors:** Harold E Bays, Debbra D Bazata, Nathaniel G Clark, James R Gavin, Andrew J Green, Sandra J Lewis, Michael L Reed, Walter Stewart, Richard H Chapman, Kathleen M Fox, Susan Grandy

**Affiliations:** 1 Louisville Metabolic and Atherosclerosis Research Center, Louisville, KY, USA; 2 Saint Luke's South Primary Care, Overland Park, KS, USA; 3 American Diabetes Association, Alexandria, VA, USA; 4 Emory University School of Medicine, Fairburn, GA, USA; 5 Midwestern Endocrinology, Overland Park, KS, USA; 6 Northwest Cardiovascular Institute, Portland, OR, USA; 7 Vedanta Research, Chapel Hill, NC, USA; 8 Center for Health Research, Geisinger Health Systems, Danville, PA, USA; 9 ValueMedics Research, LLC, Falls Church, VA, USA; 10 Strategic Healthcare Solutions, LLC, Monkton, MD, USA; 11 AstraZeneca LP, Wilmington, DE, USA

## Abstract

**Background:**

Studies derived from continuous national surveys have shown that the prevalence of diagnosed diabetes mellitus in the US is increasing. This study estimated the prevalence in 2004 of self-reported diagnosis of diabetes and other conditions in a community-based population, using data from the Study to Help Improve Early evaluation and management of risk factors Leading to Diabetes (SHIELD).

**Methods:**

The initial screening questionnaire was mailed in 2004 to a stratified random sample of 200,000 households in the US, to identify individuals, age ≥ 18 years of age, with diabetes or risk factors associated with diabetes. Follow-up disease impact questionnaires were then mailed to a representative, stratified random sample of individuals (n = 22,001) in each subgroup of interest (those with diabetes or different numbers of risk factors for diabetes). Estimated national prevalence of diabetes and other conditions was calculated, and compared to prevalence estimates from the National Health and Nutrition Examination Survey (NHANES) 1999–2002.

**Results:**

Response rates were 63.7% for the screening, and 71.8% for the follow-up baseline survey. The SHIELD screening survey found overall prevalence of self-reported diagnosis of diabetes (either type 1 or type 2) was 8.2%, with increased prevalence with increasing age and decreasing income. In logistic regression modeling, individuals were more likely to be diagnosed with type 2 diabetes if they had abdominal obesity (odds ratio [OR] = 3.50; p < 0.0001), BMI ≥28 kg/m^2 ^(OR = 4.04; p < 0.0001), or had been diagnosed with dyslipidemia (OR = 3.95; p < 0.0001), hypertension (OR = 4.82; p < 0.0001), or with cardiovascular disease (OR = 3.38; p < 0.0001).

**Conclusion:**

The SHIELD design allowed for a very large, community-based sample with broad demographic representation of the population of interest. When comparing results from the SHIELD screening survey (self-report only) to those from NHANES 1999–2002 (self-report, clinical and laboratory evaluations), the prevalence of diabetes was similar. SHIELD allows the identification of respondents with and without a current diagnosis of the illness of interest, and potential longitudinal evaluation of risk factors for future diagnosis of that illness.

## Background

Studies have shown that the prevalence of diagnosed diabetes mellitus (DM) in the US is increasing. Mokdad et al.[1], using data from the Behavioral Risk Factor Surveillance System (BRFSS), a cross-sectional telephone survey conducted by the Centers for Disease Control and Prevention and state health departments, showed that the prevalence of self-reported diagnosis of DM increased from 4.9% in 1990 to 6.5% in 1998 to 7.3% in 2000 [1,2]. Using data from four consecutive National Health and Nutrition Examination Surveys, Kanjilal et al. [3] showed a similar, significant increase in prevalence of self-reported diagnosis of DM, as well as a significant increase in total (diagnosed and undiagnosed) prevalence of DM. Further, they showed that DM prevalence increased most among persons with lower income and education levels [3].

The purpose of this study was to estimate the prevalence in 2004 of self-reported diagnosis of DM and prevalence of specific risk factors associated with diabetes in a community-based population. Additionally, individuals with risk factors for diabetes were identified to determine similarities and differences with diabetes patients. SHIELD (**S**tudy to **H**elp **I**mprove **E**arlyevaluation and management of risk factors **L**eading to **D**iabetes), the largest survey of its kind, began with an initial cross-sectional snapshot of the US population, followed by longitudinal questionnaires designed to evaluate parameters potentially related to DM, and is the first prospective study to examine select risk factors (RFs) for future diagnoses of DM. This study was also designed to provide insight into health-related knowledge, behaviors, and attitudes as well as their implications for individuals' transitions to a DM diagnosis and progression of DM treatments. The SHIELD design and methodology are described herein, along with the initial, cross-sectional results of parameters related to DM compared with findings from the National Health and Nutrition Examination Survey (NHANES) 1999–2002 data.

## Methods

SHIELD consists of three phases: 1) an initial screening, cross-sectional survey that used a general population screening questionnaire to identify cases of interest; 2) the baseline survey, in which the identified respondents were followed up longitudinally with a longer, more detailed questionnaire assessing each individual's health status, health knowledge and attitudes, and current behaviors and treatments; and 3) annual follow-up questionnaires to assess disease progression, which encompassed a) transition from at risk status to diagnosed DM, b) progression of treatment over the course of the disease, and c) the associated clinical and economic burden. Results from the first two phases are presented here. Respondents volunteered to complete the surveys without compensation.

### Screening questionnaire

The initial screening questionnaire was mailed in April 2004 to a stratified random sample of 200,000 households in the US. These households were a part of the Taylor Nelson Sofres National Family Opinion, Inc. (TNS NFO) survey panel, which is a market research firm that maintains a panel of households throughout the US for marketing and research purposes. The panel was constructed to be representative of the US population in terms of residence (including both geographic region and household area population size), age of the head of household and household income and size. The requirements for participation included being 18 years of age and having a telephone and mailing address. Random samples of households were invited to enroll in the panel, and demographic information was obtained from those who enroll (and updated every 2 years). Households who agreed to participate were invited to take part in periodic surveys. The NFO panel has been used previously to determine general population prevalence of migraine [4,5], bipolar disorder [6], disease related impairment [7-9], and depression and comorbidity in epilepsy and asthma [10].

The SHIELD screening survey used 12 questions created by an expert advisory panel to identify individuals with DM (and type of DM) or RFs associated with a diagnosis of DM. After being mailed, the screening questionnaire was completed by the head of the household who answered for up to four adult members of the household (≥18 years of age). The head of household was identified as the individual who is the panel member for the NFO panel and typically was the adult female of the household. Due to the self-administration approach, the head of household was able to consult with other adult family members and personal health records to report whether they or any adult member had been told by their doctor or nurse that they had any of the conditions.

### Risk factors (RFs) associated with diabetes mellitus

In addition to self-reported DM, the screening questionnaire included the following items: age, family history, adiposity (as measured by body mass index [BMI]) and presence of potential DM-related predictors such as abdominal obesity, hypertension, dyslipidemia, and cardiovascular disease (CVD) events. The SHIELD data were analyzed and compared with overall prevalence estimates from NHANES 1999–2002 data (which used both self-reported and laboratory values). The following factors were hypothesized to be associated with a diagnosis of DM: (1) *abdominal obesity *(waist circumference), (2) *overweight/obesity *(BMI, calculated from self-reported height and weight), (3) *cholesterol problems *(reported diagnosis of cholesterol problems of any type), (4) *hypertension *(reported diagnosis of high blood pressure), and (5) *history of CVD *("heart disease/myocardial infarction, narrow or blocked arteries, stroke, coronary artery bypass graft surgery/angioplasty/stents/surgery to clear arteries"). Respondents were provided with a measuring tape and while standing were asked to hold the tape measure loosely around their waist at the level of their navel ("belly button") to determine waist circumference.

These CVD parameters were confirmed using logistic regression analyses on the SHIELD screening data, which indicated that each RF had independent and similar predictive power for diagnosis of DM. Specific thresholds for waist circumference and BMI were determined as described below in "Data Analysis."

### Sampling for baseline survey

Once the screening questionnaire was returned, baseline (BL) follow-up disease impact questionnaires were mailed in September and October of 2004 to a representative stratified random sample of individuals based upon several subgroups of interest, which included: (1) type 1 DM (defined as reported type 1 diagnosis made at age ≤21 years plus insulin use), (2) type 2 DM (defined as reported type 2 diagnosis made at age >21 years), and (3) individuals without DM but with 0, 1, 2, 3, 4, or 5 RFs. Less than 12% of baseline survey respondents were from the same household and the correlation between responses for individuals from the same household was minimal (r < 0.03) and not statistically significant. After the BL questionnaires were returned, a subgroup of 600 responses was created from the total response pool to represent a general population sample for comparison with the DM and RF groups. This "population-based" control group sampled returns from the type 1 and type 2 DM groups and each RF level in proportions to reflect the overall prevalence of that stratum in the general population. This group was constructed by stratified random sampling (without replacement) of 600 individuals from within the disease and RF groups in proportion to their rates of occurrence in the population (as estimated from the screening study).

### Baseline (BL) questionnaire

The BL questionnaire consisted of 64 detailed questions regarding comorbidities, symptoms, and family history; medical testing; health-related quality of life, depression and health-related disruptions of normal activities; diet, exercise and other health-related behaviors; healthcare insurance coverage and resource use; and impact of health problems on work productivity.

The BL questionnaire also included several validated survey instruments including: (1) the 12-item Short Form Health Survey [11] (SF-12v2;) and the European Quality of Life (EuroQoL) EQ-5D instrument [12-15] to assess quality of life; (2) Sheehan Disability Scale [16] to assess the level of disruption felt in work, social life, and family/home life due to health problems; (3) 9-item Patient Health Questionnaire (PHQ-9) [17] to assess depression; and (4) the Work Productivity and Activity Impairment Questionnaire: General Health, version 2.0 (WPAI-GH) to assess work productivity and performance of other regular activities [18].

In addition, the questionnaire included some, but not all, questions from the following instruments: the Diet and Health Knowledge Survey (DHKS), the Press-Ganey Satisfaction Questionnaire, and the International Physical Activity Questionnaire (IPAQ), which was developed to assess health-related aspects of physical activity and sedentary behaviors [19].

### Data analysis

Estimated national prevalence of DM and each RF was calculated using SHIELD and NHANES data. Logistic regression analyses of the SHIELD screening data with diagnosis of type 2 DM as the dependent variable and sociodemographic characteristics and RFs as explanatory variables were used to identify those factors associated with being diagnosed with type 2 DM. In addition, specific thresholds for waist circumference and BMI were determined separately for men and women, using the area under receiver operating characteristic (ROC) curves to quantify sensitivity and specificity. The waist circumference or BMI value that maximized the area under the ROC curve was chosen as the threshold (point that maximized the number of people correctly classified as diabetes or not) for determining whether that RF (i.e., "abdominal obesity" or "high BMI") was present. Odds ratios around the threshold were stable. The ROC analysis was done to determine diabetes-specific thresholds for obesity (BMI) and abdominal obesity (waist circumference) rather than using the continuous variable for these factors; and the ROC model with the continuous variables was only slightly improved over the dichotomous variable. The sensitivity of the model predicting diabetes (duration ≥ 3 years) was 0.63 (range = 0.61–0.70) and specificity was 0.80 (range = 0.75–0.82). Analyses of the areas under the ROC curves for abdominal obesity found an optimal cutoff value at waist circumference ≥ 97 cm for men and waist circumference ≥ 89 cm for women. For BMI, the optimal threshold value was ≥ 28 kg/m^2 ^in both men and women.

RF levels were calculated as the unweighted number of RFs reported by each respondent on the screening questionnaire. For example, if a person reported three RFs on the screening questionnaire, they were classified into RF level 3. Most analyses of the SHIELD BL data reported here grouped individuals into cohorts with lower risk (0–2 RFs) or higher risk (3–5 RFs). Data reported here reflect the sociodemographic characteristics of the respondents to the BL survey and the occurrence rates of each RF among our respondent subgroups.

## Results

### Response rates and analyzed cohort

Of the 200,000 households that received the screening questionnaire, 127,420 households (containing a total of 211,097 adults) returned usable questionnaires, yielding a response rate of 63.7% for the screening survey. The follow-up BL survey was mailed to a total of 22,001 individuals, and 17,640 were returned, for a response rate of 80.2%. The total number of usable (i.e., no missing values) returns was 15,794 (71.8%).

### Prevalence of diabetes mellitus and risk factors

The SHIELD screening survey revealed that the overall prevalence of self-reported diagnosis of DM (either type 1 or type 2) was 8.2% for the 211,097 respondents, with increased prevalence with increasing age (Table 1). In comparison, the overall DM prevalence in NHANES for self-report was 6.5% and 9.0% for self-report and clinical and laboratory evaluations, with approximately 2.5% of the 9.0% undiagnosed.

**Table 1:** Prevalence of diabetes mellitus and risk factors in the SHIELD screening and NHANES 1999–2002 studies by age and gender

**Condition**	**Prevalence from:**					
	
	**SHIELD self-report**			**NHANES overall**		
	All	Men	Women	All	Men	Women

Diabetes mellitus	8.2	8.5	7.9	9.0	10.2	7.8
Age 18–44		3.0	2.9		4.4	3.2
Age 45–64		12.9	11.3		15.8	9.6
Age 65+		20.3	16.6		20.6	17.1

Dyslipidemia	25.8	26.9	24.8	52.9	60.2	45.8
Total cholesterol	17.1	18.0	16.3	34.8	35.9	33.7
LDL-C	9.5	10.4	8.7	13.7	15.2	12.3
HDL-C	5.0	5.5	4.5	23.7	34.1	13.4
Triglycerides	6.7	7.5	6.0	16.9	20.5	13.5

Abdominal obesity	31.5	27.9	34.8	51.4	50.8	51.9

BMI≥28 kg/m^2^	40.0	40.9	39.2	40.9	40.5	41.4

Hypertension	23.4	23.0	23.8	28.9	27.0	30.8

History of cardiovascular event	6.6	8.0	5.3	6.9	8.0	5.8

LDL-C = low-density lipoprotein cholesterol, HDL-C = high-density lipoprotein cholesterol, BMI = body mass index

The prevalence estimates for each of the 5 RFs, as found in the SHIELD and NHANES studies, are also shown in Table 1. The estimated proportions of the population with BMI ≥28 kg/m^2 ^(obese) and history of CVD events were generally similar in SHIELD and NHANES, while the prevalence of abdominal obesity and of dyslipidemia estimated in SHIELD were lower than from NHANES.

### Logistic regression analysis results

The logistic regression model of factors associated with diagnosis of type 2 DM in the screening data is shown in Table 2. Individuals were more likely to be diagnosed with type 2 DM if they had abdominal obesity (odds ratio [OR] = 3.50; p < 0.0001), or had been diagnosed with dyslipidemia (OR = 3.95; p < 0.0001) or hypertension (OR = 4.82; p < 0.0001). A BMI ≥28 kg/m^2 ^also put individuals at a significantly higher risk of being diagnosed with type 2 DM (OR = 4.04; p < 0.0001), as did a prior CV event (OR = 3.38, p < 0.0001).

**Table 2:** Logistic regression analysis of risk factors associated with being diagnosed with type 2 diabetes mellitus

	Odds Ratio	95% Confidence Interval	P value
**Risk Factors**			
Abdominal Obesity	3.50	[3.31, 3.71]	<.0001
BMI ≥28 kg/m2	4.04	[3.83, 4.27]	<.0001
Diagnosed Dyslipidemia	3.95	[3.75, 4.15]	<.0001
Diagnosed Hypertension	4.82	[4.58, 5.07]	<.0001
History of Cardiovascular event	3.38	[3.19, 3.58]	<.0001
**Demographic Variables**			
Male Gender	1.18	[1.13, 1.24]	<.0001
Age (10-year increase)	1.35	[1.33, 1.37]	<.0001
Race			<.0001
Black/African American	1.67	[1.53, 1.82]	
Asian/Pacific Islander	0.82	[0.63, 1.05]	
American Indian/Eskimo	1.33	[1.00, 1.77]	
Other	0.84	[0.66, 1.06]	
Ethnicity			<.0001
Not Spanish/Hispanic	1.00		
Spanish/Hispanic	0.98	[0.85, 1.14]	
Household Income ($)			<.0001
< 22,500	2.59	[2.39, 2.80]	
22,500–39,999	1.84	[1.69, 2.00]	
40,000–59,999	1.49	[1.36, 1.62]	
60,000–89,999	1.35	[1.23, 1.47]	
Household Size			<.0001
1 Member	2.30	[2.06, 2.57]	
2 Members	1.99	[1.79, 2.21]	
3 Members	1.49	[1.33, 1.67]	
4 Members	1.06	[0.94, 1.20]	
Household Area Population Size			<.0001
<100,000	1.18	[1.10, 1.27]	
100,000–499,999	1.11	[1.03, 1.19	
500,000–1,999,999	1.09	[1.03, 1.17]	
Census Region			<.0001
New England	1.09	[0.96, 1.14]	
Middle Atlantic	1.06	[0.96, 1.16]	
East North Central	1.08	[0.98, 1.18]	
West North Central	0.99	[0.88, 1.12]	
South Atlantic	1.25	[1.15, 1.37]	
East South Central	1.28	[1.14, 1.44]	
West South Central	1.25	[1.13, 1.38]	
Mountain	0.92	[0.82, 1.05]	

Reference groups: Gender = female; Race = White; Spanish = Not Spanish; Region = Pacific; HH income = $90,000+; HH size = 5+; HH Area Population size = 2,000,000+.

Age was entered as a continuous variable.

BMI = body mass index; HH = household

After adjusting for other factors in the model, men were more likely to be diagnosed with type 2 DM than women (OR = 1.18; p < 0.001). African Americans were at a higher risk for being diagnosed with type 2 DM than were individuals who responded white for race (OR = 1.67). Lower household income and increased age were also associated with increased odds of type 2 DM diagnosis.

### Sociodemographic data

Sociodemographic characteristics of the study population sample (n = 600) and individuals with diagnosed type 1 (n = 368) and type 2 DM (n = 3,898), and 0–2 (n = 5,295) and 3–5 RF (n = 5,400) groups are shown in Additional File 1. US census data are also included for comparison. Younger age groups, African Americans, and Asian Americans were generally under-represented in the study population, compared with US census data.

With some exceptions, in general, the sociodemographic characteristics of the population sample group and US census data were similar to those seen in the 0–2 RF group. In contrast, at least with regard to age, income, and household size, the 3–5 RF group had sociodemographic characteristics more similar to the type 2 DM group. The type 1 DM group tended to be younger and the type 2 DM group tended to be older than the population sample. In summary, the 3–5 RF and type 2 DM groups tended to be older, and to have lower incomes and smaller household size compared to the 0–2 RF group, population control sample, and US census. The lower income and smaller household size relationship for the 3–5 RF and type 2 DM groups remained even with age stratification.

### RF proportions

The proportion of respondents with individual RFs within each subgroup of interest are shown in Table 3. The type 1 DM group had similar proportions of abdominal obesity and BMI ≥28 as the population sample and the 0–2 RF groups. Both the type 1 and type 2 DM subgroups had a higher likelihood of being diagnosed with dyslipidemia, hypertension, and CVD events (perhaps in part related to more intensive medical evaluations once being diagnosed with DM) compared to the population sample and 0–2 RF group. The type 2 DM group and the 3–5 RF group had generally similar individual RF proportions and a similar average number of risk factors. Approximately 78% of the type 2 DM group had 3–5 risk factors. Finally, within each subgroup, CVD was the least frequently reported RF.

**Table 3:** Proportion of respondents with risk factors and by diabetes group

		Type 1	Type 2		
Risk Factor	Population Sample	Diabetes Mellitus	Diabetes Mellitus	0–2 RFs	3–5 RFs
	(n = 600)	(n = 368)	(n = 3,898)	(n = 5,295)	(n = 5,400)
Men, %	38.7%	38.9%	42.3%	34.5%	43.1%
Abdominal Obesity	41.8%	44.9%	86.6%	48.0%	95.9%
18–44 years	35.4%	44.0%	88.3%	50.9%	97.8%
45–64 years	43.0%	46.0%	88.6%	44.7%	96.8%
≥ 65 years	53.6%	50.0%	83.4%	47.1%	93.8%
BMI ≥28 kg/m^2^	34.7%	36.7%	76.3%	35.7%	88.0%
18–44 years	32.9%	38.5%	87.3%	42.9%	95.9%
45–64 years	38.9%	34.4%	82.8%	33.4%	92.7%
≥ 65 years	29.1%	25.0%	64.2%	19.9%	79.0%
Dyslipidemia	26.3%	43.5%	72.7%	19.6%	81.2%
18–44 years	12.6%	40.9%	60.2%	9.7%	74.5%
45–64 years	33.2%	46.1%	74.6%	26.9%	80.1%
≥ 65 years	41.8%	75.0%	74.5%	32.0%	85.5%
Hypertension	20.8%	30.4%	67.6%	12.6%	76.2%
18–44 years	7.3%	24.1%	47.8%	4.0%	60.9%
45–64 years	26.6%	39.8%	68.2%	15.1%	76.3%
≥ 65 years	38.2%	62.5%	73.2%	32.0%	83.5%
Cardiovascular					
Event	7.5%	16.0%	30.2%	3.8%	36.7%
18–44 years	1.2%	8.2%	11.7%	1.9%	14.2%
45–64 years	5.7%	25.8%	25.6%	3.0%	28.0%
≥ 65 years	25.5%	87.5%	42.0%	11.5%	58.2%
					
Number of Risk					
Factors					
0–2 risk factors	80.1%	67.2%	21.7%	100%	0%
3–5 risk factors	19.9%	32.8%	78.3%	0%	100%
Mean (SD)	1.3 (1.3)	1.7 (1.5)	3.3 (1.2)	1.2 (0.8)	3.8 (0.7)

BMI = body mass index

## Discussion

The SHIELD design allowed for a very large, community-based sample with broad demographic representation of the population of interest. The use of the TNS NFO household survey panel also resulted in a high response rate for a written survey. The completion of the questionnaire in the home setting allowed for thoughtful answers and for time to check records and medications for accurate reporting. In addition, the respondents could work at their pace, and there was no interviewer bias. Any sampling bias due to demographics can be measured and adjusted for in the final analysis. Finally, the survey allowed the identification of respondents without a current diagnosis of the illness of interest, and potential longitudinal evaluation of RFs for future diagnosis of that illness.

SHIELD consists of a cross-sectional, and then 5-year longitudinal observational study of individuals with or at risk for DM. A large number of the cross-sectional, screening questionnaires were sent and a high return rate was achieved, providing a sample that was generally representative of the overall US population. The BL survey was designed to provide a more detailed view of DM and other health conditions in a large sample that will be followed over 4 subsequent annual surveys. The overall response rate for the BL survey (80%) was also quite high for a large, mailed survey.

Demographically, the population sample was generally similar to US census data, indicating that the SHIELD results are representative and generalizable to the US population. The population sample and the 0–2 RF groups were also similar to each other. One important result from this survey was the similarity of the 3–5 RF and the type 2 DM groups. Results from SHIELD reveal that the mean number of RFs increases with age, as does the likelihood of being diagnosed with DM. The proportion of individual RFs was highest in the type 2 DM and 3–5 RF groups, with somewhat higher percentages for each of the five individual RFs in the 3–5 RF group as compared with the type 2 DM group. The 3–5 RF group has not been diagnosed with diabetes but they appear similar to the type 2 DM group, which is not explained by age stratification. This finding may indicate the importance of managing the risk factors in the 3–5 RF group to delay or prevent diabetes. Importantly, in follow-up surveys, it may be possible to determine which RFs in those persons without DM best correlate to a future diagnosis of DM. The similarity in prevalence rates of DM between SHIELD and NHANES confirm that surveys like SHIELD, with self-reported diagnoses, acquire data approximating surveys that also include clinical and laboratory evaluations.

The lower prevalence of dyslipidemia and abdominal obesity in SHIELD versus NHANES may be largely due to the use of laboratory data (cholesterol levels) and interviewer measurement in NHANES compared with only self-report in SHIELD. For diagnoses that are dependent on laboratory evaluations, particularly when more than one laboratory parameter is used to define a specific diagnosis (such as dyslipidemia), self-report surveys may underestimate the true prevalence of these diagnoses. Multivariate analysis indicated that smaller household size, population size and the US South were independently associated with a higher likelihood of type 2 DM diagnosis. These associations may be due to differences in diet, lifestyle habits, access to medical and/or diabetes care and limited family support.

It should be noted that panel data have some limitations. For example, only a small percentage (5%–8%) of consumers invited to participate in the NFO panel elected to do so, leading to the possibility of bias. Household panels tend to under-represent the very wealthy and very poor segments of the population, and do not include military or institutionalized individuals. However, these limitations are true for most random sampling and clinically based methodologies as well.

Another limitation is that data collected by self-reported surveys cannot always be directly compared to clinical and laboratory surveys, such as NHANES data. This is especially true in trying to assess clustering of CVD risk factors.

Currently, further analyses of the screening and BL survey data are in progress, to investigate differences in health attitudes and behaviors between survey respondents with type 1 and type 2 DM and those with 3–5 RFs. It is anticipated that the long-term, longitudinal data from SHIELD will allow for continued clarification of predictors of being tested for or being diagnosed with DM. In addition, it is possible that SHIELD data may help identify those RFs (and health attitudes and behaviors) that are most predictive of transitioning from one stage of DM to the next (disease progression) and from one stage of DM treatment to the next (treatment progression).

## Conclusion

When comparing results from the SHIELD screening survey to those from NHANES 1999–2002 (self-report and clinical and laboratory evaluations), the prevalence of DM was similar. In addition, the prevalence of DM observed in SHIELD increased with higher ages and lower income levels. Multivariable analyses of the SHIELD baseline survey data found that abdominal obesity, higher BMI, and diagnosis of cholesterol problems, hypertension or CVD were each independently associated with higher likelihood of type 2 DM diagnosis. Other factors positively associated with type 2 DM diagnosis were increased age, black race, and decreased household income level.

SHIELD is an ongoing self-reported survey study that began with a cross-sectional questionnaire, followed by targeted longitudinal surveys, and is the largest survey of its kind. Because a large number of questionnaires were sent with a high return rate achieved, the SHIELD survey achieved a large sample that is representative of the overall US population. SHIELD provides information that is unique from data more commonly reported, especially regarding the longitudinal follow-up on a large sample, which may allow correlation of RFs with the future diagnosis of DM.

## Competing interests

Dr. Harold Bays, in almost two decades of clinical research, has served as a Clinical Investigator for (and has received research grants from) pharmaceutical companies such as Abbott, Alteon, Arena, AstraZeneca, Aventis, Bayer, Boehringer Ingelheim, Boehringer Mannheim, Bristol Myers Squibb, Eli Lilly, Esperion, Fujisawa, Ciba Geigy, GelTex, Glaxo, Genentech, Hoechst Roussel, KOS, Kowa, Lederle, Marion Merrell Dow, Merck, Merck Schering Plough, Miles, Novartis, Parke Davis, Pfizer, Pliva, Purdue, Reliant, Roche, Rorer, Regeneron, Sandoz, Sankyo, Sanofi, Searle, Shionogi, Schering Plough, SmithKline Beecham, Takeda, TAP, UpJohn, Upsher Smith, Warner Lambert, and Wyeth-Ayerst. He has also served as a consultant, speaker, and/or advisor to and for pharmaceutical companies such as Arena, AstraZeneca, Aventis, Bayer, Bristol Myers Squibb, KOS, Merck, Merck Schering Plough, Metabasis Therapeutics, Microbia, Novartis, Nicox, Ortho-McNeil, Parke Davis, Pfizer, Roche, Sandoz, Sankyo, Sanofi Aventis, Shering Plough, SmithKline Beacham, Takeda, UpJohn, and Warner Lambert. Dr. James Gavin has served as a consultant to Sanofi-Aventis, Merck, Novartis, Eli Lilly, LifeScan, and Mannkind; on advisory boards for AstraZeneca, Novartis, Eli Lilly, and Novo Nordisk; on speakers' bureaus for Eli Lilly, Novartis, Sanofi-Aventis; and as a director for Amylin. Ms. Bazata and Drs. Clark, Green, Lewis and Stewart serve on advisory boards for AstraZeneca LP. Drs. Michael Reed, Richard Chapman and Kathleen Fox received funding for research and consulting from AstraZeneca LP. Dr. Susan Grandy is an employee of AstraZeneca LP. SHIELD, the SHIELD Advisory Board, and the preparation of this manuscript were supported by funding from AstraZeneca LP.

## Authors' contributions

HB chairs the SHIELD Advisory Board, and helped to draft the manuscript. DDB, NGC, JRG, AJG, SJL, MLR, and WS were members of the SHIELD Advisory Board, which participated in the design and ongoing direction of this study. RHC participated in the design and coordination of this study, analysis of the results, and drafting of the manuscript. KF participated in analysis of the results and drafting of the manuscript. SG participated in the design and coordination of this study and drafting of the manuscript. All the authors have read and approved the final manuscript.

## Pre-publication history

The pre-publication history for this paper can be accessed here:



## Supplementary Material

Additional file 1 Sociodemographic characteristics of respondents to the SHIELD baseline survey (unweighted). Click here for file
