# Supplementary material for: Prevalence of self-reported diagnosis of diabetes mellitus and associated risk factors in a national survey in the US population: SHIELD (Study to Help Improve Early evaluation and management of risk factors Leading to Diabetes)
Source: BMC Public Health. 2007 Oct 3;7:277. doi: 10.1186/1471-2458-7-277 (PMC2222165; doi:10.1186/1471-2458-7-277)
Supplement: Additional file 1 — Sociodemographic characteristics of respondents to the SHIELD baseline survey (unweighted). [file 1471-2458-7-277-S1.doc]

| **Sociodemographic characteristics of respondents to the SHIELD baseline survey (unweighted)** | | | | | | | |
| --- | --- | --- | --- | --- | --- | --- | --- |
|  |  | US Census* | Population Sample + (n=600) | Type 1^ (n=368) | Type 2^  (n=3,898) | 0–2 RFs^ (n=5,295) | 3–5 RFs^  (n=5,400) |
| Gender | |  |  |  |  |  |  |
|  | Men | 48.2% | 38.7% | 38.9% | 42.3% | 34.5% | 43.1% |
|  | 18-44 years |  | 35.4% | 37.9% | 33.9% | 31.2% | 34.0% |
|  | 45-64 years |  | 37.3% | 39.1% | 40.5% | 36.4% | 41.4% |
|  | >65 years |  | 49.1% | 62.5% | 47.2% | 39.8% | 47.9% |
|  | Women | 51.8% | 61.3% | 61.1% | 57.7% | 65.5% | 56.9% |
|  | 18-44 years |  | 64.6% | 62.1% | 66.1% | 68.8% | 66.0% |
|  | 45-64 years |  | 62.7% | 60.9% | 59.5% | 63.6% | 58.6% |
|  | >65 years |  | 50.9% | 37.5% | 52.8% | 60.2% | 52.1% |
| Age |  |  |  |  |  |  |  |
|  | 18–24 | 12.9% | 6.7% | 9.5% | 0.1% | 7.6% | 0.6% |
|  | 25–34 | 18.5% | 15.3% | 25.5% | 2.6% | 17.3% | 5.2% |
|  | 35–44 | 20.7% | 19.0% | 28.0% | 9.9% | 23.2% | 11.4% |
|  | 45–54 | 18.9% | 23.5% | 22.8% | 21.0% | 21.5% | 20.6% |
|  | 55–64 | 12.9% | 17.2% | 12.0% | 28.1% | 13.6% | 24.5% |
|  | 65–74 | 8.5% | 9.8% | 1.6% | 21.8% | 9.3% | 20.4% |
|  | 75+ | 7.6% | 8.5% | 0.5% | 16.5% | 7.5% | 17.2% |
| Race |  |  |  |  |  |  |  |
|  | White | 82.5% | 86.7% | 90.2% | 84.8% | 88.4% | 88.3% |
|  | 18-44 years |  | 87.4% | 87.5% | 79.3% | 86.5% | 83.6% |
|  | 45-64 years |  | 86.9% | 94.5% | 83.9% | 89.1% | 87.3% |
|  | >65 years |  | 84.5% | 100% | 87.8% | 90.3% | 90.8% |
|  | Black/African American | 11.5% | 6.5% | 2.7% | 9.6% | 5.5% | 7.0% |
|  | 18-44 years |  | 6.1% | 3.9% | 12.3% | 6.6% | 10.1% |
|  | 45-64 years |  | 7.0% | 0.8% | 10.2% | 5.1% | 8.3% |
|  | >65 years |  | 6.4% | 0% | 8.0% | 4.1% | 4.7% |
|  | Asian/Pacific Islander | 4.2% | 0.5% | 0.8% | 0.8% | 1.2% | 0.2% |
|  | 18-44 years |  | 0.4% | 1.3% | 1.6% | 1.7% | 0.4% |
|  | 45-64 years |  | 0.4% | 0% | 0.7% | 0.9% | 0.2% |
|  | >65 years |  | 0.9% | 0% | 0.7% | 0.4% | 0.1% |
|  | American Indian/Eskimo | 0.7% | 0.8% | 1.9% | 0.7% | 0.7% | 0.7% |
|  | 18-44 years |  | 0.8% | 1.7% | 0.8% | 0.6% | 1.1% |
|  | 45-64 years |  | 1.2% | 2.3% | 1.0% | 0.8% | 0.8% |
|  | >65 years |  | 0% | 0% | 0.1% | 0.4% | 0.4% |
|  | Other | 1.2% | 1.8% | 1.6% | 0.9% | 1.5% | 0.9% |
|  | No Answer | NA | 3.7% | 2.7% | 3.2% | 2.8% | 2.9% |
| Ethnicity** | |  |  |  |  |  |  |
|  | Spanish/Hispanic | 12.3% | 3.0% | 3.5% | 2.9% | 3.1% | 2.0% |
|  | 18-44 years |  | 3.7% | 4.7% | 6.2% | 4.7% | 4.5% |
|  | 45-64 years |  | 3.3% | 1.6% | 3.0% | 2.2% | 1.9% |
|  | >65 years |  | 0.9% | 0% | 1.7% | 1.1% | 1.0% |
|  | Not Spanish/Hispanic | 87.7% | 92.3% | 93.8% | 91.3% | 92.6% | 92.2% |
|  | 18-44 years |  | 94.3% | 92.7% | 90.3% | 92.8% | 91.8% |
|  | 45-64 years |  | 91.8% | 95.3% | 92.4% | 93.8% | 93.8% |
|  | >65 years |  | 89.1% | 100% | 90.2% | 88.6% | 90.3% |
|  | No Answer | NA | 4.7% | 2.7% | 5.8% | 4.3% | 5.8% |
| Annual Household Income | |  |  |  |  |  |  |
|  | <$22,500 | 19.6% | 19.7% | 21.7% | 30.1% | 17.8% | 25.6% |
|  | 18-44 years |  | 18.3% | 20.7% | 30.0% | 18.2% | 23.4% |
|  | 45-64 years |  | 16.4% | 24.2% | 28.0% | 14.1% | 21.7% |
|  | >65 years |  | 30.0% | 12.5% | 32.9% | 25.6% | 32.4% |
|  | $22,500–$39,999 | 19.3% | 18.2% | 20.1% | 22.5% | 18.9% | 21.2% |
|  | 18-44 years |  | 19.9% | 23.7% | 26.5% | 21.5% | 23.8% |
|  | 45-64 years |  | 14.8% | 12.5% | 19.7% | 14.2% | 18.0% |
|  | >65 years |  | 21.8% | 37.5% | 24.7% | 21.4% | 24.0% |
|  | $40,000–$59,999 | 18.8% | 18.2% | 20.1% | 16.7% | 19.0% | 17.9% |
|  | 18-44 years |  | 20.7% | 22.4% | 18.9% | 20.7% | 22.1% |
|  | 45-64 years |  | 14.3% | 17.2% | 16.8% | 17.4% | 16.7% |
|  | >65 years |  | 20.9% | 0% | 15.8% | 17.3% | 16.7% |
|  | $60,000–$89,999 | 20.2% | 20.5% | 17.1% | 15.8% | 21.1% | 17.4% |
|  | 18-44 years |  | 19.5% | 19.4% | 14.8% | 20.8% | 18.4% |
|  | 45-64 years |  | 23.4% | 11.7% | 17.1% | 23.1% | 20.1% |
|  | >65 years |  | 16.4% | 37.5% | 14.4% | 17.2% | 13.3% |
|  | $90,000+ | 22.1% | 23.5% | 20.9% | 14.9% | 23.2% | 17.9% |
|  | 18-44 years |  | 21.5% | 13.8% | 9.9% | 18.7% | 12.4% |
|  | 45-64 years |  | 31.1% | 34.4% | 18.4% | 31.1% | 23.5% |
|  | >65 years |  | 10.9% | 12.5% | 12.2% | 18.4% | 13.4% |
| Household Size | |  |  |  |  |  |  |
|  | 1 Person | 13.9% | 13.8% | 18.5% | 22.8% | 14.0% | 23.2% |
|  | 18-44 years |  | 7.3% | 18.1% | 14.0% | 8.5% | 10.4% |
|  | 45-64 years |  | 17.2% | 20.3% | 18.6% | 14.0% | 18.9% |
|  | >65 years |  | 20.9% | 0% | 31.0% | 30.0% | 34.1% |
|  | 2 Persons | 33.9% | 39.2% | 34.2% | 44.9% | 35.5% | 44.6% |
|  | 18-44 years |  | 24.0% | 27.6% | 26.5% | 21.6% | 23.0% |
|  | 45-64 years |  | 40.6% | 43.8% | 43.2% | 42.7% | 44.4% |
|  | >65 years |  | 70.0% | 75.0% | 53.1% | 58.9% | 54.2% |
|  | 3 Persons | 19.4% | 19.5% | 20.7% | 15.6% | 19.2% | 14.4% |
|  | 18-44 years |  | 24.8% | 20.7% | 22.6% | 23.2% | 24.7% |
|  | 45-64 years |  | 19.7% | 20.3% | 18.7% | 19.4% | 16.5% |
|  | >65 years |  | 7.3% | 25.0% | 9.3% | 7.4% | 7.7% |
|  | 4 Persons | 18.1% | 19.5% | 16.0% | 9.5% | 17.4% | 10.3% |
|  | 18-44 years |  | 30.5% | 20.7% | 21.8% | 25.8% | 25.5% |
|  | 45-64 years |  | 16.4% | 8.6% | 11.1% | 13.4% | 11.5% |
|  | >65 years |  | 1.8% | 0% | 3.5% | 1.9% | 1.9% |
|  | 5+ Persons | 14.7% | 8.0% | 10.6% | 7.2% | 13.9% | 7.4% |
|  | 18-44 years |  | 13.4% | 12.9% | 15.2% | 20.8% | 16.4% |
|  | 45-64 years |  | 6.1% | 7.0% | 8.4% | 10.4% | 8.7% |
|  | >65 years |  | 0% | 0% | 3.1% | 1.8% | 2.2% |
| Household Area Population Size | |  |  |  |  |  |  |
|  | <100,000 | 13.6% | 14.8% | 14.4% | 14.4% | 15.0% | 14.8% |
|  | 18-44 years |  | 15.0% | 11.2% | 18.1% | 16.5% | 18.4% |
|  | 45-64 years |  | 14.3% | 20.3% | 14.9% | 13.6% | 15.3% |
|  | >65 years |  | 15.5% | 12.5% | 12.4% | 13.2% | 12.8% |
|  | 100,000–499,999 | 16.6% | 16.8% | 18.8% | 16.6% | 16.9% | 17.6% |
|  | 18-44 years |  | 18.7% | 21.6% | 18.9% | 17.9% | 20.2% |
|  | 45-64 years |  | 18.0% | 13.3% | 17.2% | 15.4% | 18.0% |
|  | >65 years |  | 10.0% | 25.0% | 15.0% | 17.1% | 15.6% |
|  | 500,000–1,999,999 | 22.5% | 21.5% | 23.6% | 24.1% | 23.1% | 22.8% |
|  | 18-44 years |  | 22.8% | 25.4% | 24.2% | 23.0% | 25.5% |
|  | 45-64 years |  | 21.3% | 21.9% | 25.6% | 24.5% | 22.4% |
|  | >65 years |  | 19.1% | 0% | 22.0% | 20.0% | 22.2% |
|  | 2,000,000+ | 47.2% | 46.8% | 43.2% | 45.0% | 45.1% | 44.8% |
|  | 18-44 years |  | 43.5% | 41.8% | 38.8% | 42.6% | 36.0% |
|  | 45-64 years |  | 46.3% | 44.5% | 42.2% | 46.5% | 44.3% |
|  | >65 years |  | 55.5% | 62.5% | 50.5% | 49.7% | 49.3% |
| Census Region | |  |  |  |  |  |  |
|  | New England | 5.1% | 4.5% | 3.8% | 5.2% | 4.9% | 4.8% |
|  | 18-44 years |  | 4.9% | 3.0% | 4.5% | 4.5% | 3.6% |
|  | 45-64 years |  | 3.7% | 5.5% | 4.6% | 5.7% | 4.8% |
|  | >65 years |  | 5.5% | 0% | 6.1% | 4.3% | 5.6% |
|  | Middle Atlantic | 14.4% | 15.2% | 13.3% | 14.8% | 14.0% | 14.9% |
|  | 18-44 years |  | 17.1% | 10.8% | 12.9% | 14.6% | 13.5% |
|  | 45-64 years |  | 15.2% | 18.0% | 14.4% | 14.0% | 15.2% |
|  | >65 years |  | 10.9% | 12.5% | 15.9% | 12.4% | 15.1% |
|  | East North Central | 15.9% | 17.2% | 16.8% | 16.6% | 17.6% | 18.3% |
|  | 18-44 years |  | 18.7% | 19.8% | 16.8% | 17.9% | 17.1% |
|  | 45-64 years |  | 12.3% | 11.7% | 17.4% | 17.4% | 18.5% |
|  | >65 years |  | 24.5% | 12.5% | 15.5% | 16.9% | 18.4% |
|  | West North Central | 6.8% | 7.7% | 7.3% | 6.8% | 8.0% | 7.1% |
|  | 18-44 years |  | 8.1% | 8.2% | 6.6% | 8.1% | 6.9% |
|  | 45-64 years |  | 7.8% | 5.5% | 7.5% | 8.4% | 7.3% |
|  | >65 years |  | 6.4% | 12.5% | 5.9% | 6.4% | 6.7% |
|  | South Atlantic | 18.8% | 17.2% | 16.8% | 21.3% | 17.7% | 20.0% |
|  | 18-44 years |  | 14.6% | 15.5% | 20.9% | 16.7% | 20.6% |
|  | 45-64 years |  | 19.3% | 18.0% | 19.7% | 17.4% | 18.5% |
|  | >65 years |  | 18.2% | 37.5% | 23.3% | 21.4% | 21.4% |
|  | East South Central | 6.0% | 4.3% | 4.3% | 6.6% | 6.7% | 6.3% |
|  | 18-44 years |  | 3.7% | 3.0% | 8.8% | 7.1% | 9.6% |
|  | 45-64 years |  | 5.7% | 7.0% | 7.0% | 6.3% | 6.8% |
|  | >65 years |  | 2.7% | 0% | 5.5% | 6.0% | 4.4% |
|  | West South Central | 11.1% | 10.8% | 11.4% | 10.8% | 9.9% | 10.6% |
|  | 18-44 years |  | 10.6% | 12.5% | 10.1% | 9.8% | 10.5% |
|  | 45-64 years |  | 11.1% | 8.6% | 10.9% | 9.2% | 11.3% |
|  | >65 years |  | 10.9% | 25.0% | 10.9% | 11.5% | 10.1% |
|  | Mountain | 6.5% | 9.2% | 10.9% | 5.5% | 7.0% | 5.6% |
|  | 18-44 years |  | 9.3% | 9.9% | 6.8% | 7.2% | 6.4% |
|  | 45-64 years |  | 9.0% | 13.3% | 5.9% | 7.0% | 5.2% |
|  | >65 years |  | 9.1% | 0% | 4.6% | 6.2% | 5.6% |
|  | Pacific | 15.5% | 14.0% | 15.2% | 12.4% | 14.2% | 12.4% |
|  | 18-44 years |  | 13.0% | 17.2% | 12.5% | 14.1% | 11.8% |
|  | 45-64 years |  | 16.0% | 12.5% | 12.5% | 14.4% | 12.4% |
|  | >65 years |  | 11.8% | 0% | 12.2% | 15.0% | 12.7% |

+ Control group based upon a stratified random sampling of 600 of the 17,640 “baseline” returned responses, adjusted to have proportional risk factor prevalence similar to the general population.

^ Derived from the baseline returned responses. The risk factor groups did not have diabetes mellitus. Some of the baseline responders were omitted due to the definition of diabetes mellitus, which included medication and age restrictions. For example, a patient self-reporting having “type 1 diabetes mellitus” who reported having the onset of diabetes mellitus >= 21 years of age and/or not treated with insulin was excluded from the analysis.

*United States Census Bureau. 2003 Annual Supplement to the Current Population Survey: Census Bureau Resident Population Estimates of the United States.

** Hispanics are asked to indicate their origin in the question on Hispanic origin, not in the question on race, following the US federal statistical system, in which ethnic origin is considered to be a separate concept from race. People of Hispanic origin may be any race.

NA = not applicable
